# Effect of Hormone Replacement Therapy on Cardiovascular Outcomes: A Meta-Analysis of Randomized Controlled Trials

**DOI:** 10.1371/journal.pone.0062329

**Published:** 2013-05-08

**Authors:** Dicheng Yang, Jing Li, Zhongxiang Yuan, Xu Liu

**Affiliations:** 1 Department of Cardiovascular Surgery, Shanghai First People’s Hospital affiliated to Shanghai Jiao Tong University, Shanghai, China; 2 Department of Cardiology, Shanghai Chest Hospital affiliated to Shanghai Jiao Tong University, Shanghai, People’s Republic of China; University of Valencia, Spain

## Abstract

**Background:**

Hormone replacement therapy (HRT) is widely used to controlling menopausal symptoms and prevent adverse cardiovascular events. However, the benefit and risk of HRT on cardiovascular outcomes remains controversial.

**Methodology and Principal Findings:**

We systematically searched the PubMed, EmBase, and Cochrane Central Register of Controlled Trials databases for obtaining relevant literature. All eligible trials reported on the effects of HRT on cardiovascular outcomes. We did a random effects meta-analysis to obtain summary effect estimates for the clinical outcomes with use of relative risks calculated from the raw data of included trials. Of 1903 identified studies, we included 10 trials reporting data on 38908 postmenopausal women. Overall, we noted that estrogen combined with medroxyprogesterone acetate therapy as compared to placebo had no effect on coronary events (RR, 1.07; 95%CI: 0.91–1.26; P = 0.41), myocardial infarction (RR, 1.09; 95%CI: 0.85–1.41; P = 0.48), stroke (RR, 1.21; 95%CI: 1.00–1.46; P = 0.06), cardiac death (RR, 1.19; 95%CI: 0.91–1.56; P = 0.21), total death (RR, 1.06; 95%CI: 0.81–1.39; P = 0.66), and revascularization (RR, 0.95; 95%CI: 0.83–1.08; P = 0.43). In addition, estrogen therapy alone had no effect on coronary events (RR, 0.93; 95%CI: 0.80–1.08; P = 0.33), myocardial infarction (RR, 0.95; 95%CI: 0.78–1.15; P = 0.57), cardiac death (RR, 0.86; 95%CI: 0.65–1.13; P = 0.27), total mortality (RR, 1.02; 95%CI: 0.89–1.18; P = 0.73), and revascularization (RR, 0.77; 95%CI: 0.45–1.31; P = 0.34), but associated with a 27% increased risk for incident stroke (RR, 1.27; 95%CI: 1.06–1.53; P = 0.01).

**Conclusion/Significance:**

Hormone replacement therapy does not effect on the incidence of coronary events, myocardial infarction, cardiac death, total mortality or revascularization. However, it might contributed an important role on the risk of incident stroke.

## Introduction

Hormone replacement therapy (HRT) has been available to postmenopausal women for more than 60 years. Although the use of HRT therapy for control of moderate to severe menopausal symptoms and prevention of osteoporosis is well established, its long term use for cardiovascular disease prevention in postmenopausal women remains controversial. Previous observational studies have shown HRT was associated with a reduction of 30–50% in the risk of coronary events [1]–[3], however, data from randomized controlled trials failed to show the benefit of HRT for cardiovascular outcomes, even found that HRT could induce stroke events [4]–[5]. Which makes interpretation of the results difficult for clinicians and has further restricted its applocation in clinical prevention.

The reason for such inconsistent evidence could be that individual trial might have been underpowered to show clinical benefit, especially if event rates were lower than were expected and improve risk factors for control group; duration of treatment was shorter than was need to show a clinical benefit; differences in the clinical characteristics of the study populations; and methodologic limitations of observational studies may identify women for whom postmenopausal HRT confers a higher or lower risk of cardiovascular events. Which makes interpretation of the results difficult for clinicians and has further restricted its application in clinical prevention.

Previous meta-analysis [6] failed to show the benefit of HRT on cardiovascular outcomes and concluded HRT could increases the risk of stroke in postmenopausal women. Several large-scale randomized controlled trials investigating the effect of HRT on cardiovascular outcomes have been completed. For a better understanding of the effect of HRT on cardiovascular outcomes, data from these trials need to be evaluated to formulate a conclusion. We therefore conducted a systematic review and meta-analysis of pooled data from randomized controlled trials to evaluate the possible effect of HRT on cardiovascular outcomes in postmenopausal women.

## Methods

### Data Sources, Search Strategy, and Selection Criteria

We gathered data from randomized controlled trials that were designed to assess the effects of HRT on cardiovascular outcomes in postmenopausal women. Furthermore, we also stipulated that any trials comparing HRT with placebo needed to follow up participants in both treatment group identically to avoid systematic error and resultant bias. We systenatically searched the English literature to identify all relevant randomized controlled trials regardless of publication status (published, in press, and in progress), and to assess the effects of HRT on cardiovascular outcomes. Relevant trials were identified using the following procedure:

1. Electronic searches: We searched the PubMed, EmBase, and the Cochrane Central Register of Controlled Trials electronic databases for articles published through Nov. 10, 2012, using “hormone replacement therapy” OR “oestrogen replacement therapy” OR “estradiol” OR “progesterone” OR “HRT” AND “cardiovascular” AND “human” AND “English” AND “randomized controlled trials” as the search terms. All reference lists from reports on non-randomized controlled trials were searched manually for additional eligible studies.
2. Other sources: We searched ongoing randomized controlled trials, which had been registered as completed but not yet published, in http://www.ClinicalTrials.gov websites for information on registered randomized controlled trials. In addition, the relevant review and meta-analyses regarding the role of HRT for postmenopausal women were examined for potential inclusive trials. Medical subject headings, population and intervention were used to identify relevant trials initially.

The literature search was undertaken independently by 2 reviewers (DY and JL) with a standardized approach, any inconsistencies between these 2 reviewers were settled by the third reviewer (XL) until a consensus was reached. All completed randomized controlled trials evaluating the effects of HRT on cardiovascular outcomes. Studies were eligible for inclusion if: (1). The study was a randomized controlled trials; (2). Reporting at least 1 outcome of major vascular events; (3). The trials published in English. This review was conducted and reported according to the PRISMA (Preferred Reporting Items for Systematic Reviews and Meta-Analysis) Statement issued in 2009 (Table S1) [7].

### Data Collection and Quality Assessment

Two authors (DY and JL) independently extracted and collected data using a standardized data-extraction protocol. Any inconsistent was settled by group discussion, after this, the primary author (ZY) made the final decision. The data collected included: first author or study group name, publication year, number of patients, mean age, pre-existing disease, intervention regimes, duration of follow-up, and the number of outcome for both treatment group. The outcomes investigated included coronary events, stroke, myocardial infarction, cardiac death, and any possible adverse events. We measured the quality of the trials included in this study with the Jadad score [8], which basis of randomization, concealment of treatment allocation, blinding, completeness of follow-up, and use of intention-to-treat analysis.

### Statistical Analysis

To standardize reporting of our results, relative risks (RRs) and 95% confidence intervals (CIs) were calculated from raw data of every included trial. We assessed the effect of HRT versus placebo on cardiovascular outcomes with a random-effects model with Mantel–Haenszel statistics, which assumes that the true underlying effect varies between studies. Heterogeneity of treatment effects between studies was investigated visually by scatter plot analysis and statistically by the heterogeneity I^2^ statistic [9]–[10]. We used risk estimates obtained with random-effects models instead of fixed-effects models, because this approach provides a more conservative assessment of the average effect size [11]–[12]. Subgroup analysis was conducted based on the number of patients, mean age, pre-existing disease, and interventions. Egger’s test [13] was used to check for potential publication bias. All reported P values were two-sided and P values of less than 0.05 were regarded as significant for all included trials. Statistical analyses were carried out using software STATA (Version 10.0).

## Results

**Figure 1:**
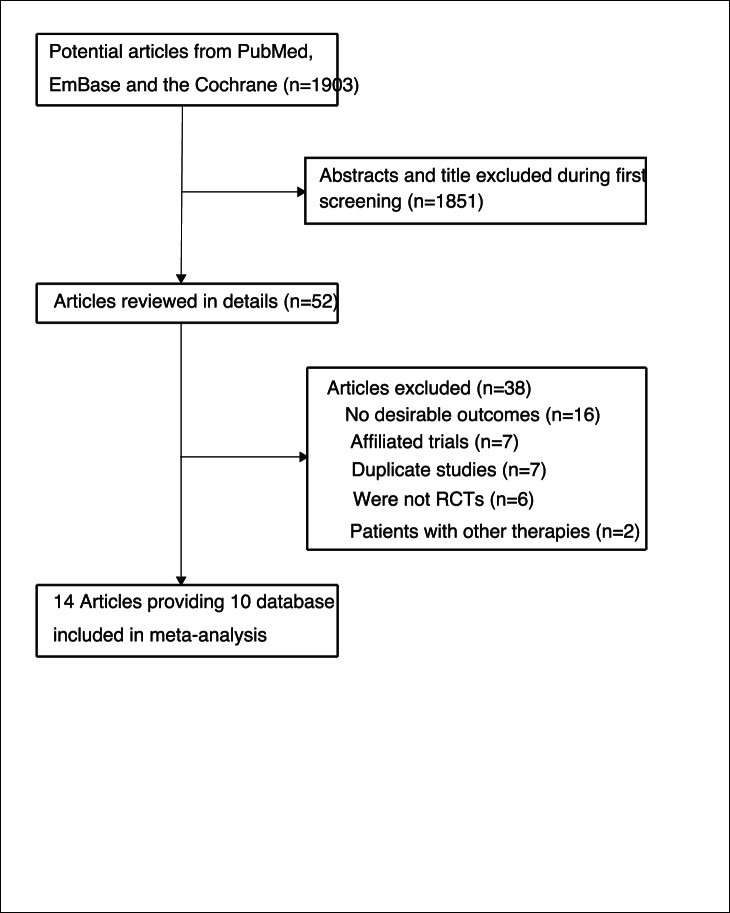
Flow diagram of the literature search and trials selection process.

We screened the titles and abstracts of 1903 potentially eligible studies, and 1851 of them were excluded after a preliminary review of searches. The remaining 52 studies were retrieved for detailed assessment. Of these, fourteen trials [4], [5], [14]–[25] providing ten database met the inclusion criteria, which consisted of data of 38908 postmenopausal women (Figure 1 and Figure S1). 6 of these [5], [15], [16], [18], [24], [25] evaluated conjugated equine estrogen combined with medroxyprogesterone acetate therapy comparing with placebo and the remaining four study [20]–[23] evaluated estrogen therapy comparing with placebo. Table 1 summarizes the characteristics of these trials and the important information of the included indivuduals. The size of sample ranged from 222 to 16608, with a mean of 3891, the mean study follow-up ranged from 1 to 6.8 years, with a mean of 3.3 years. 4 trials evaluated individuals with previous cardiovascular disease or stroke, 2 trials assessed individuals with cardiovascular risk factors, and remaining four trials evaluated healthy women. Although the included trials scarcely reported on the key indicators of trial quality, the quality of the included trials were also evaluated according to the pre-defined criteria using the Jadad score. Overall, two of included trials [20], [23] scored 5, seven trials [5], [14], [16], [18], [21], [22], [25] scored 4, and the remaining one trial [24] scored 3.

**Table 1:** Design and characteristic of trials included in our meta-analysis.

Source	Publicationyear	No. of patients	Mean age, y	Status	Intervention	Control	Follow-up (year)	Jadad score
The ERA Study Group [5]	2000	309	65.8	Coronary artery disease	0.625 mg of conjugated equine estrogen; 0.625 mgof conjugated equine estrogen plus 2.5 mg of medroxyprogesterone acetate	Placebo	3.2	4
The HERS Research Group [4], [14], [15]	2002	2763	67.0	Coronary heart disease	0.625 mg of conjugated estrogen and 2.5 mg of medroxyprogesterone acetate	Placebo	4.1	4
The WAVE Study Group [16]	2002	423	65.5	15–75% coronary stenosis	0.625 mg of conjugated equine estrogen plus2.5 mg of medroxyprogesterone acetate	Placebo	2.8	4
The Women’s Health Initiative Investigators [17]–[19]	2003	16608	63.2	Heathy women	0.625 mg of conjugated equine estrogen plus2.5 mg of medroxyprogesterone acetate	Placebo	5.2	4
The WEST Study Group [20]	2001	664	71.5	Recently had an ischemic stroke or transient ischemic attack	1 mg of estradiol-17*β*	Placebo	2.8	5
The ESPRIT Team [21]	2002	1017	62.6	Previous myocardial infarction	2 mg of oestradiol valerate	Placebo	2	4
The Women’s Health Initiative Steering Committee [22]	2004	10739	63.6	Previous hysterectomy	0.625 mg of conjugated equine estrogen	Placebo	6.8	4
The EPAT Study Group [23]	2001	222	61.5	low-density lipoprotein cholesterol levels of 3.37 mmol/L or greater	1 mg of estradiol-17*β*	Placebo	2	5
P Veerus [24]	2006	1778	58.7	Healthy women	0.625 mg of conjugated equine estrogen plus 2.5/5 mg of medroxyprogesterone acetate	Placebo or notreatment	3.4	3
The WISDOM Team [25]	2007	4385	63.3	Healthy women	0.625 mg of conjugated equine estrogen plus 2.5/5 mg of medroxyprogesterone acetate	Placebo	1	4

**Figure 2:**
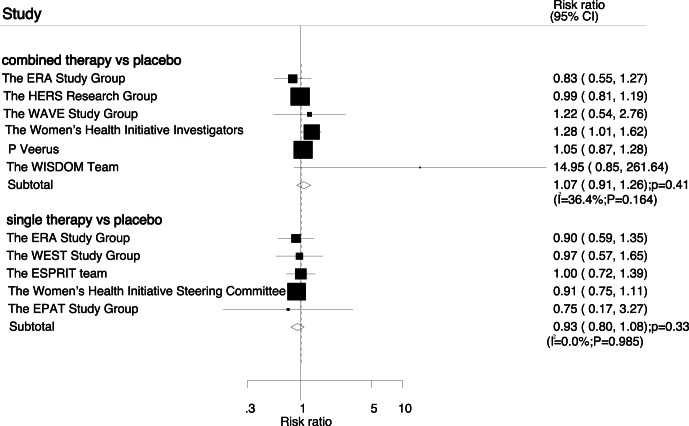
Effect of hormone replacement therapy on risk of coronary events.

Data for the effect of combined therapy on coronary events were available from 6 trials, which included 26166 postmenopausal women and reported 1068 coronary events. Overall, there was no evidence to show that combined therapy could reduce the risk of coronary events (RR, 1.07; 95%CI: 0.91–1.26; P = 0.41, Figure 2). Although heterogeneity was observed in the magnitude of the effect across the trials included. However, after sequential exclusion of each trial from all pooled analysis, the results were similar when excluding any specific trial. Additionally, five trials reported the effect of estrogen therapy alone on coronary events, which including 12847 participants and recorded 619 coronary events. Overall, estrogen therapy alone reduced the risk of coronary events by 7%, but was not associated with a statistically significant decrease in the risk of coronary events (RR, 0.93; 95%CI: 0.80–1.08; P = 0.33; without evidence of heterogeneity, Figure 2).

**Figure 3:**
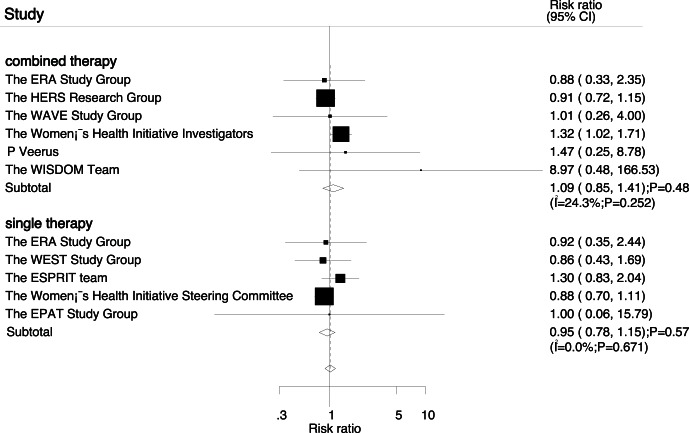
Effect of hormone replacement therapy on risk of myocardial infarction.

Data for the effect of combined therapy on myocardial infarction were available from 6 trials, which included 26166 postmenopausal women and reported 517 events of myocardial infarction. Overall, reduction in the risk of myocardial infarction with combined therapy was not statistically significant (RR, 1.09; 95%CI: 0.85–1.41; P = 0.48; with unimportant heterogeneity, Figure 3). Additionally, five trials reported the effect of estrogen therapy alone on myocardial infarction, which including 12847 participants and recorded 406 events of myocardial infarction. No effect of estrogen therapy alone on the risk of myocardial infarction was observed (RR, 0.95; 95%CI: 0.78–1.15; P = 0.57; without evidence of heterogeneity, Figure 3).

**Figure 4:**
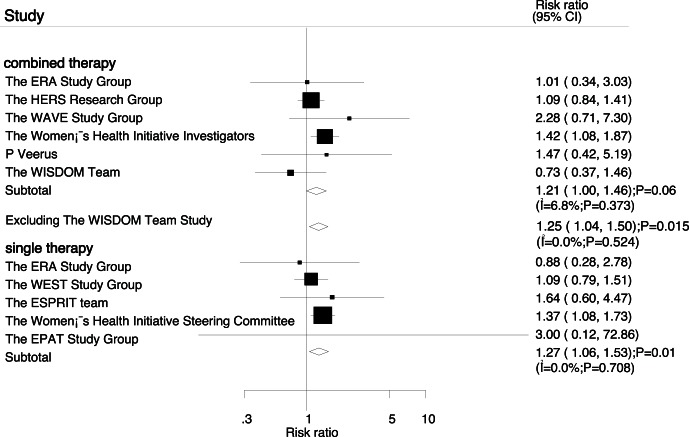
Effect of hormone replacement therapy on risk of stroke.

Data for the effect of combined therapy on stroke were available from 6 trials, which included 26166 postmenopausal women and reported 495 stroke events. Overall, combined therapy increased the risk of stroke by 21% when compared with placebo, but this difference was not associated with a statistically significant (RR, 1.21; 95%CI: 1.00–1.46; P = 0.06, with unimportant heterogeneity, Figure 4). According to a sensitivity analysis, we excluded the WISDOM Study [24], this trial specifically with less than 1 year of follow-up, which always with low incident of stroke. After this, we concluded that combined therapy was associated with a statistically significant increased the risk of stroke when compared with placebo (RR, 1.25; 95%CI: 1.04–1.50; P = 0.01, without evidence of heterogeneity, Figure 4). Additionally, five trials reported the effect of estrogen therapy alone on stroke, which including 12847 participants and recorded 423 stroke events. Overall, we noted that estrogen therapy alone increased the risk of stroke by 27% when compared with placebo (RR, 1.27; 95%CI: 1.06–1.53; P = 0.01, without evidence of heterogeneity, Figure 4).

Table 2 shows the effects of HRT (combined therapy or estrogen therapy alone) on cardiac death, total death, and revascularization as compared to placebo. Overall, no significant differences were identified between the effect of HRT and placebo, whether participants received combined therapy or estrogen therapy alone.

**Table 2:** Summary of the relative risks of all adverse outcomes assessed.

Outcomes	Group	Intervention group	placebo	RR and 95% CI	P value	Heterogeneity (%)	P value for heterogeneity
Coronary events	Combined therapy vs placebo	561/13294	507/12872	1.07 (0.91–1.26)	0.41	36	0.16
	Estrogen vs placebo	296/6371	323/6476	0.93 (0.80–1.08)	0.33	0	0.99
Myocardial infarction	Combined therapy vs placebo	273/13294	244/12872	1.09 (0.85–1.41)	0.48	24	0.25
	Estrogen vs placebo	196/6371	210/6476	0.95 (0.78–1.15)	0.57	0	0.67
Stroke	Combined therapy vs placebo	274/13294	221/12872	1.21 (1.00–1.46)	0.06	7	0.37
	Estrogen vs placebo	237/6371	186/6476	1.27 (1.06–1.53)	0.01	0	0.71
Cardiac death	Combined therapy vs placebo	113/10200	94/9803	1.19 (0.91–1.56)	0.21	0	0.93
	Estrogen vs placebo	90/6371	106/6476	0.86 (0.65–1.13)	0.27	0	0.80
Total death	Combined therapy vs placebo	256/11016	237/10609	1.06 (0.81–1.39)	0.66	8	0.35
	Estrogen vs placebo	379/6260	375/6365	1.02 (0.89–1.18)	0.73	0	0.63
Revascularization	Combined therapy vs placebo	412/10200	424/9803	0.95 (0.83–1.08)	0.43	0	0.51
	Estrogen vs placebo	19/211	26/216	0.77 (0.45–1.31)	0.34	0	0.72

Subgroup analyses were conducted for coronary events, and stroke to evaluate the effect of HRT on cardiovascular outcomes in some specific population. Overall, we noted that estrogen therapy was associated with a significant increase in the risk for stroke, when trials with more than 1000 individuals, mean age of included trials was less than 65, and participants were health women or hysterectomy (Table 3). No other significant differences were detected between the effect of HRT and placebo, based on additional subset factors.

**Table 3:** Subgroup analysis for the effect of hormone replacement therapy on coronary events, and stroke.

Outcomes	Group	Sugroup	Relative risk (RR) and 95%CI	P value	heterogeneity (%)	P value for heterogeneity
**Coronary events**	**Combined therapy vs placebo**	**Number of patients**				
		>1000	1.10 (0.91–1.34)	0.31	52	0.10
		<1000	0.90 (0.62–1.31)	0.58	0	0.41
		**Mean age**				
		>65	0.97 (0.82–1.15)	0.70	0	0.66
		<65	1.19 (0.89–1.59)	0.25	58	0.09
		**Pre-existing disease**				
		vascular risk factors	0.97 (0.82–1.15)	0.70	0	0.66
		Health women or hysterectomy	1.19 (0.89–1.59)	0.25	58	0.09
	**Estrogen therapy alone vs placebo**	**Number of patients**				
		>1000	0.93 (0.79–1.11)	0.42	0	0.63
		<1000	0.91 (0.66–1.26)	0.58	0	0.94
		**Mean age**				
		>65	0.92 (0.67–1.28)	0.63	0	0.81
		<65	0.93 (0.78–1.10)	0.40	0	0.86
		**Pre-existing disease**				
		vascular risk factors	0.95 (0.76–1.20)	0.69	0	0.97
		Health women or hysterectomy	0.91 (0.75–1.11)	0.35	–	–
**Stroke**	**Combined therapy vs placebo**	**Number of patients**				
		>1000	1.18 (0.93–1.50)	0.17	27	0.25
		<1000	1.48 (0.67–3.30)	0.33	0	0.32
		**Mean age**				
		>65	1.12 (0.88–1.43)	0.36	0	0.47
		<65	1.19 (0.77–1.85)	0.43	36	0.21
		**Pre-existing disease**				
		vascular risk factors	1.12 (0.88–1.43)	0.36	0	0.47
		Health women or hysterectomy	1.19 (0.77–1.85)	0.43	36	0.21
	**Estrogen therapy alone vs placebo**	**Number of patients**				
		>1000	1.38 (1.10–1.74)	0.006	0	0.73
		<1000	1.08 (0.79–1.48)	0.61	0	0.77
		**Mean age**				
		>65	1.07 (0.78–1.47)	0.66	0	0.72
		<65	1.39 (1.10–1.74)	0.005	0	0.84
		**Pre-existing disease**				
		vascular risk factors	1.12 (0.83–1.52)	0.44	0	0.77
		Health women or hysterectomy	1.37 (1.08–1.73)	0.009	–	–

We used Egger’s test [13] to check for potential publication bias, which showed no evidence of publication bias for the outcomes of coronary events (combined therapy vs placebo: P = 0.739; estrogen therapy vs placebo: P = 0.310), stroke (combined therapy vs placebo: P = 0.357; estrogen therapy vs placebo: P = 0.172), and myocardial infarction (combined therapy vs placebo: P = 0.977; single therapy: P = 0.519).

## Discussion

Recently, evidence from large-scale randomized controlled trials [17]–[19] has shown that HRT is not significantly more effective than placebo in reducing the rate of coronary events. In addition, the risk of life-threatening stroke events has been shown to increase with HRT. This comprehensive systematic review included 38908 individuals in 10 trials with a broad range of baseline characteristics. The results of our study suggest that HRT does not effect on the incidence of coronary events, myocardial infarction, cardiac death, total mortality, or revascularization. In addition, estrogen therapy alone significantly increased the risk of stroke events when compared with placebo.

The relationship between HRT and coronary heart disease were described initially by observational studies [3], [26], however, the effect of HRT in reducing the risk of coronary events has not been confirmed by randomized controlled trials. The reason for this could be as follows: observational studies and randomized controlled trials may be at least partially attributable to differences in the clinical characteristics of the study populations, including differences in age, years since menopause; furthermore, the possibility that these associations mere reflect the effects of the diet or lifestyle on cardiovascular disease rates cannot be ruled out, which led us may overestimate the effect of this relationship. Therefore, we carried out a systematic review and meta-analysis based on randomized controlled trials to explain the possible effect of HRT on coronary events, and any possible drug-related adverse events.

Our main findings are in contrast with the findings of previous study [6], and also support the conclusions that HRT does not effect on the risk of coronary events, myocardial infarction, cardiac death, total mortality, or revascularization. The reason for these could be that although estrogen therapy reduces plasma levels of LDL cholesterol and increases levels of HDL cholesterol, could improves endothelial vascular function, however, it also has adverse physiological effects, including increasing the plasma levels of triglycerides, small dense LDL particles, C-reactive protein, and so on [27]–[30]. Therefore, although HRT may have direct beneficial effects on cardiovascular outcomes, these effects may be reduced or balanced by the adverse physiological effects.

HRT play an important role on the risk of incident stroke when compared with placebo. The small but persistent increase in systolic blood pressure in women receiving hormore replacement therapy is one possible contributor to this effect because relatively small differences in systolic blood pressure have been positively associated with differences in stroke and cardiovascular disease rates [31]–[32].

There was no significant differences between HRT and placebo in the relative risk for total mortality, the reason for this could be that the use of HRT resulted in a higher rate of nonfatal stroke and a suggestion of more severe functional deficits, which may have contributed to a high mortality rate. Hence, the effect of HRT on total mortality may be reduced or balanced by drug-related adverse events.

No significant differences in the relative risk of coronary events, myocardial infarction, cardiac death, and revascularization. The reason for the absence of an effect of HRT could be that HRT has proinflammatory effects that offset its beneficial effects. Previous epidemiologic studies [3] and clinical trials [5] concluded that women reveived HRT always with higher levels of C-reactive protein. A important meta-analysis [33] already suggested that elevated levels of C-reactive protein, and the underlying inflammation were associated with the risk of cardiovascular events in women.

Previous meta-analysis [6], [34] has illustrated that the risk of coronary events is not significantly reduced using HRT when compared with placebo, moreover, the risk of stroke was significantly increased by HRT compared with placebo, these conclusions were similar to our current study. Our subgroup analysis studied important factors which could affect the interpretation of our data, these conclusions were similar to previous meta-analysis. The results of this meta-analysis are promising because we updata the results and resolve the uncertain efficacy of HRT in postmenopausal women. Furthermore, we also conducted subgroup analysis to evaluate the potential effect of HRT on cardiovascular outcomes in some specific subsets.

The limitations of our study are as follows: (1) Inherent assumptions made for all meta-analysis, because the analysis uses pooled data either from published papers or provided by individual study authors, individual data and original data were not available, which prevented us doing more detailed relevant analysis and obtaining more comprehensive results. (2) Different follow-up times could have affected our conclusions about the association between HRT and coronary outcomes. Therefore, we just gave a relative result by comparing HRT with placebo and provided a synthetic and comprehensive review.

Despite the limitations of our study, the results suggest that HRT shoud not be recommeded for cardiovascular disease prevention in postmenopausal women. Therefore, in future study, it is important to focus on healthy individuals for primary prevention of cardiovascular disease, and to combined other drugs to provide an optimal therapy that minimizes adverse events in postmenopausal women. We suggest that the ongoing trials be improved in the following ways: (1) the adverse events in clinical trials should be recorded and reported normatively, so that the side-effects of any treatment can be evaluated in future trials. (2) the role of treatment duration and dosage should be investigated in more detail to explore optimal dose and duration of treatment.

## Supporting Information

Figure S1 PRISMA Flowchart.
(DOC)

Table S1 PRISMA Checklist.
(DOC)
