# Supplementary figures and images for: Effect of Hormone Replacement Therapy on Cardiovascular Outcomes: A Meta-Analysis of Randomized Controlled Trials
Source: PLoS One. 2013 May 8;8(5):e62329. doi: 10.1371/journal.pone.0062329 (PMC3648543; doi:10.1371/journal.pone.0062329)

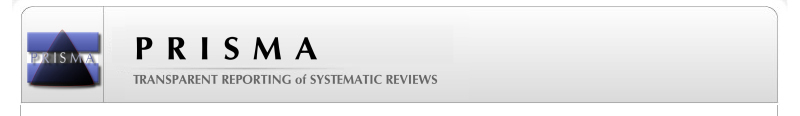
**PRISMA 2009 Flow Diagram**

**Screening**

**Included**

**Eligibility**

**Identification**

Supplement: Figure S1 — PRISMA Flowchart. (DOC) [file pone.0062329.s001.doc]
